# A novel algorithm-driven hybrid simulation learning method to improve acquisition of endotracheal intubation skills: a randomized controlled study

**DOI:** 10.1186/s12871-021-01557-6

**Published:** 2022-02-08

**Authors:** Aida Mankute, Laima Juozapaviciene, Justinas Stucinskas, Zilvinas Dambrauskas, Paulius Dobozinskas, Elizabeth Sinz, David L. Rodgers, Mantas Giedraitis, Dinas Vaitkaitis

**Affiliations:** 1 Department of Emergency Medicine, Lithuanian University of Health Sciences, Kaunas, Lithuania; 2 Department of Anaesthesiology, Lithuanian University of Health Sciences, Kaunas, Lithuania; 3 Department of Orthopaedics Traumatology, Lithuanian University of Health Sciences, Kaunas, Lithuania; 4 Department of Surgery, Lithuanian University of Health Sciences, Kaunas, Lithuania; 5 Department of Disaster Medicine, Lithuanian University of Health Sciences, Kaunas, Lithuania; 6 Department of Anesthesiology and Perioperative Medicine, Penn State Health Milton S. Hershey Medical Center, Hershey, USA; 7 Medical Simulation Center, Penn State Health Milton S. Hershey Medical Center, Hershey, PA USA

**Keywords:** HybridLab, Endotracheal intubation, Learning outcomes, Self-directed learning, Peer-to-peer simulation

## Abstract

**Background:**

Simulation-based training is a clinical skill learning method that can replicate real-life situations in an interactive manner. In our study, we compared a novel hybrid learning method with conventional simulation learning in the teaching of endotracheal intubation.

**Methods:**

One hundred medical students and residents were randomly divided into two groups and were taught endotracheal intubation. The first group of subjects (control group) studied in the conventional way via lectures and classic simulation-based training sessions. The second group (experimental group) used the hybrid learning method where the teaching process consisted of distance learning and small group peer-to-peer simulation training sessions with remote supervision by the instructors. After the teaching process, endotracheal intubation (ETI) procedures were performed on real patients under the supervision of an anesthesiologist in an operating theater. Each step of the procedure was evaluated by a standardized assessment form (checklist) for both groups.

**Results:**

Thirty-four subjects constituted the control group and 43 were in the experimental group. The hybrid group (88%) showed significantly better ETI performance in the operating theater compared with the control group (52%). Further, all hybrid group subjects (100%) followed the correct sequence of actions, while in the control group only 32% followed proper sequencing.

**Conclusions:**

We conclude that our novel algorithm-driven hybrid simulation learning method improves acquisition of endotracheal intubation with a high degree of acceptability and satisfaction by the learners’ as compared with classic simulation-based training.

## Background

The science of education is constantly looking for novel ways to improve the learning process. In medical studies, teaching students’ practical skills that can be readily and safely applied in clinical practice is a major goal. Simulation-based training is an established, important learning tool in medical education facilitating the acquisition of necessary competences and has been reported to be more effective and superior to problem-oriented learning [1, 2]. However, there are some disadvantages. Acquiring robust skills that can be applied in clinical practice requires multiple repetitions of the skill in a simulated environment, which takes considerable time and teaching resources. It has been reported that a minimum of 50 practices are needed for successful endotracheal intubation skill acquisition [3].

Our learning lab (HybridLab® at the Lithuanian University of Health Science [LUHS]) has developed a learning method designed to use simulation resources more efficiently and to empower learners to exercise effective small group peer-to-peer simulation training sessions with direct or remote supervision of the instructors, either synchronously or after the video review (asynchronously). This method offers a well-structured and standardized learning pathway, which encompasses studies on an e-learning platform, peer-to-peer hands-on training sessions in the skill lab or simulation classes using carefully elaborated learning algorithms, direct feedback by peers, and assessment by a remotely working instructor [4–6]. Mobile technologies and algorithm-driven learning facilitates peer-to-peer learning, offers an opportunity to save time and human resources in the simulation centers, creates unique possibilities for learners to explore the benefits of autonomous and self-regulated learning, and develop new feedback and peer assessment techniques as well as leadership qualities. Interactive algorithms used in the learning process guide the novice learners in a step-by-step manner, helping them to create a well-structured mental pathway for decision making and/or execution of the procedure, obviating any possible learning mistakes.

The hybrid learning system is supported by two theoretical frameworks. Ericsson’s [7] Deliberate Practice model reinforces the concept that practice with feedback is essential to improvement. This feedback needs to include both self-reflective feedback as well as feedback from others (e. g. faculty, peers) as compared to a standard of performance. Bloom’s [8] Mastery Learning model includes breaking larger tasks into smaller elements and perfecting the performance of each element before moving on to the next element.

This hybrid training method was developed at the Crisis Research Center at LUHS. To our knowledge, this hybrid model is novel, with several published studies documenting its characteristics [5, 6]. There have been no similar studies published on endotracheal intubation skills.

Our hypothesis is that the hybrid learning technique is superior to conventional simulation-based learning techniques currently used in teaching airway management skills in the Anesthesia module to medical students at the postgraduate and/or resident training level. Therefore, the aim of our study was to compare our hybrid learning method with a classic simulation-based training for teaching endotracheal intubation skills.

## Methods

### Study population and design

The study was approved by the The Lithuanian University of Health Sciences Bioethics.

Commission no. BEC-MF-442. All procedures performed in studies involving human participants were in accordance with the ethics standards of the institutional and national research committee and with the 1964 Helsinki Declaration and its later amendments or comparable ethics standards. All the participants, both patients and students/residents, gave written informed consent. This was a randomized experimental design with 78 medical students in their 5th year course in the Anesthesia module and 22 first-year residents of Emergency Medicine and Anesthesiology. The study was carried out at LUHS from May 1st through June 1st, 2019. Enrollment was performed on a consecutive manner. All students/residents in Anesthesia module were included during the study period. Randomization was performed prior to the teaching using a random number generator. Initially 50 students/residents were allocated to each study group (a) conventional simulation-based learning group (control group) and (b) the hybrid learning group (experimental group). Subjects who had previous similar practical skill training or experience were excluded from the study, as well as participants who failed to complete the course.

**Fig. 1:**
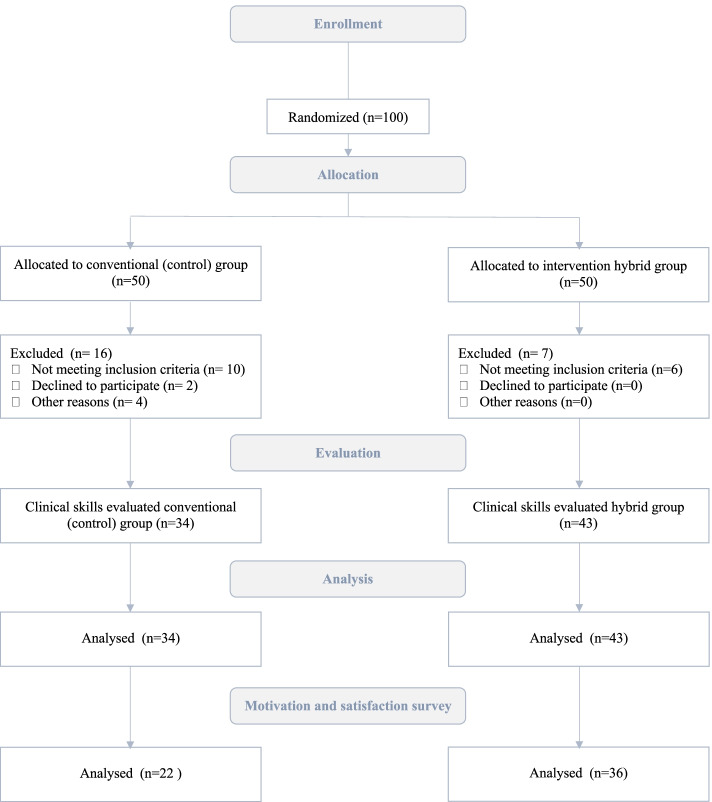
Study flowchart

The participants in both groups studied the principles of safe airway management and practical skills of endotracheal (orotracheal) intubation. The learning aim and goals were identical for both groups, and the content of theoretical knowledge, including ETI steps and skill set were aligned. Sixteen participants in the conventional simulation group and 7 participants in the hybrid group were excluded from the analysis. In total, 77 participants remained in the study (Fig. 1).

The conventional simulation-based training group teaching process consisted of lectures and practical skills with hands-on training sessions. The total duration of the course was 6 h (two sessions of 3 h). During the first simulation-based training session, the correct procedure for endotracheal intubation was demonstrated by the teacher on a manikin, which was followed by participants performing the intubation on the manikin under the teacher’s supervision. This three-hour session was taught to 10 participants simultaneously. During the second session the same 10 participants could practice intubation independently without a teacher for another 3 h.

**Fig. 2:**
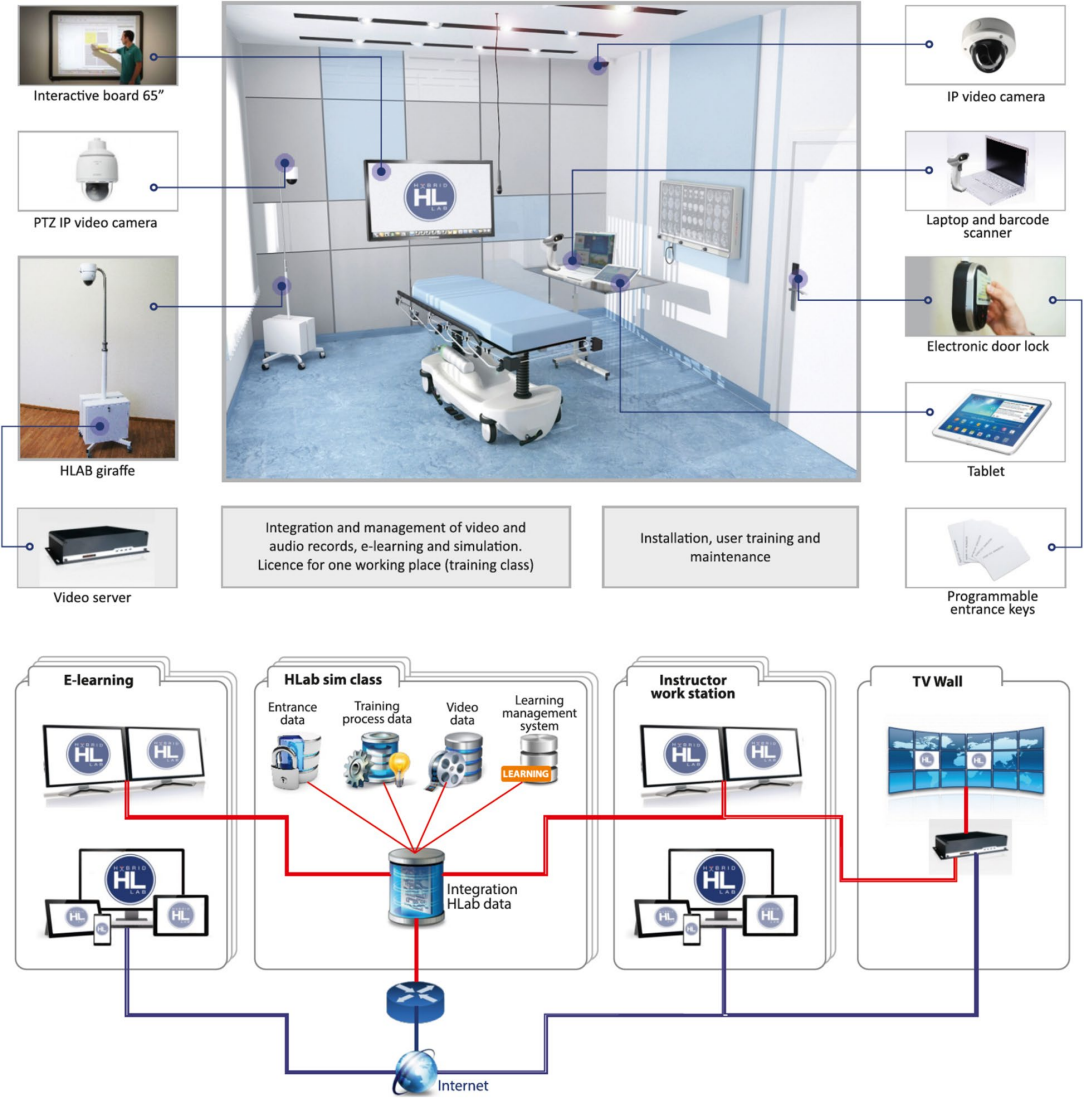
HybridLab classroom

**Fig. 3:**
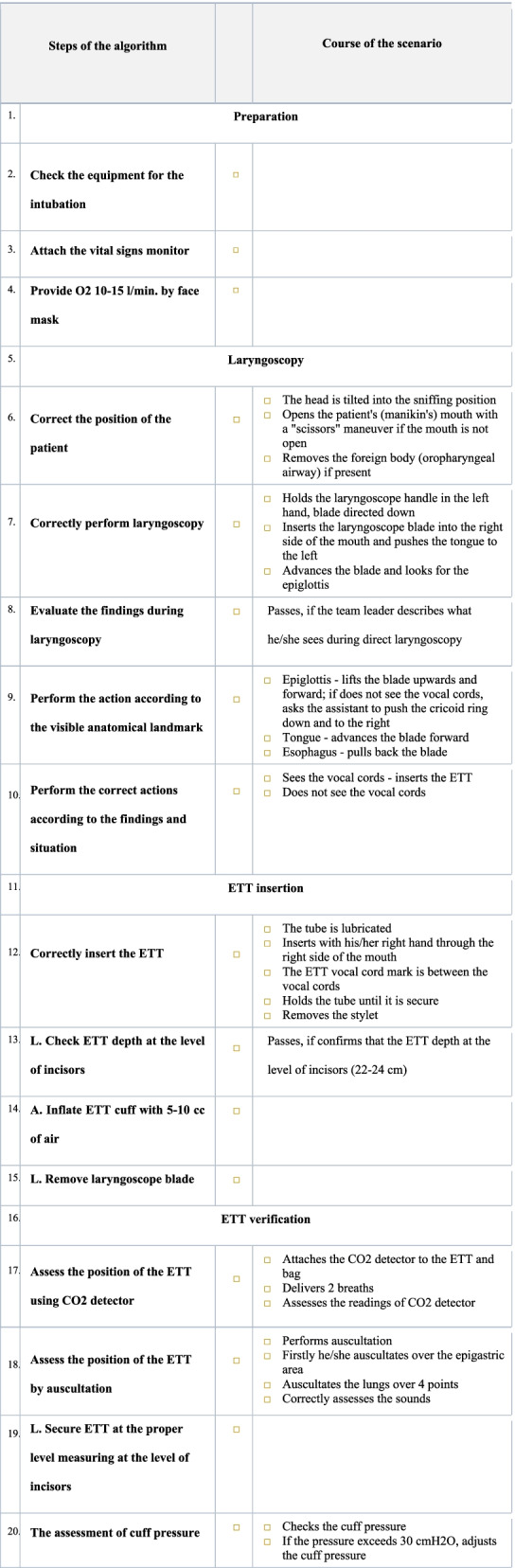
Study checklist

**Fig. 4:**
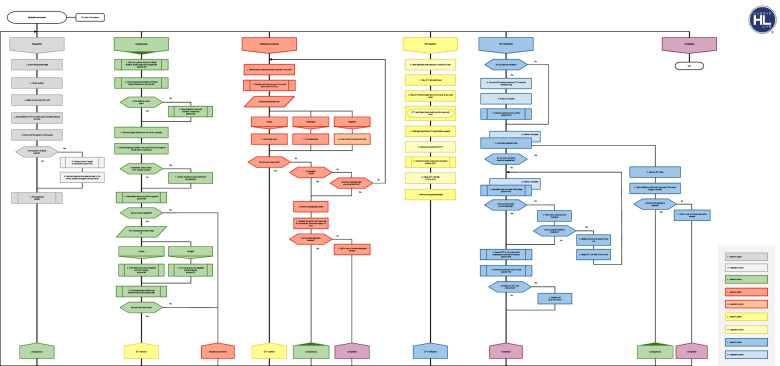
Study algorithm

The hybrid group initially studied the necessary theoretical material, lectures, videos, and algorithms on a virtual learning environment (which is comparable to Moodle and other widely used distance learning platforms). The students continued with the practical training only after passing an online test containing 10 questions with a perfect score. After the individual theoretical preparation, the subjects organized themselves into the groups of three for peer-to-peer practical skills training sessions in the HybridLab (Fig. 2). The pre-planned estimated duration of the skills training in the hybrid group was 6 h (two sessions of 3 h) and was the same as in the conventional group. However, the HybridLab subjects were encouraged to adapt the learning time based on their needs and practice at their own pace as they had 24/7 access to the training lab and did not need to coordinate the timing with the technician and/or instructor. When learning practical skills, the subjects were using the same manikin and equipment as in the conventional simulation-based training group. However, this group had the supplementary support of hand-held tablets containing proprietary educational software, electronic scenarios and checklists for clinical situation assessment and feedback (Fig. 3) as well as the learning algorithms (Fig. 4), which enabled simulation and training in the absence of a technician or instructor. Training sessions were video recorded on the cameras that were installed in the lab. The records of both the training and evaluation scenarios were viewed and evaluated remotely by the teachers. Standardized checklists were used for the evaluation, which were the same as what the subjects were using in training class. The evaluation and feedback of the teacher was provided by email.

Subjects were then asked to evaluate the course. Out of 77 participants, 58 (22 in conventional and 36 in hybrid group) completed the post-course survey. Two questions were asked regarding confidence and satisfaction: 1. After learning, I think I will pass the test successfully. 2. I enjoyed studying in this module. Each question had a 5-point Likert-type scale to evaluate the degree of student’s agreement to a given statement, as follows: score 5 for *strongly agree*, 4 for *agree*, 3 for *undecided*, 2 for *disagree*, and 1 for *strongly disagree*.

For the final evaluation of both groups, endotracheal intubation procedures were performed on real patients in the operation room (OR) under the direct supervision of the clinical teacher (anesthesiologist). The only patients who were selected for the intubation were grade I and II according to the American Society of Anesthesiologists (ASA) [9], non-obese and with good visualization of soft palate and uvula (Mallampati classification) [10]. Patients’ selection was done by one senior anesthesiology consultant who was blinded to student/resident randomization. To ensure the patient’s safety, each procedure step was closely monitored and if necessary, immediately corrected by an anesthesiologist. In addition, each step of the procedure was evaluated using the standardized assessment form (checklist). The checklist consisted of 16 steps (Fig. 3) and comprised the following parts: preparation for intubation, laryngoscopy, insertion of endotracheal tube, and verification of the placement of the endotracheal tube. The task was evaluated by the anesthesiologist who was blinded to the group the subject was enrolled in.

The sequence of endotracheal intubation procedure actions was assessed and compared between the groups. Also, participants were divided into groups according to the scope of activities performed (~ > 76%, 75–51%, 50–26, < 25%). Additionally, the satisfaction and confidence responses were compared between the groups.

### Statistical analysis

With an assumption that the difference in learning outcomes will be 30% better in the HybridLab group as compared with the conventional trained group, and with a statistical power of 0.8 and a risk of 0.05 for type-1 error, 30 participants were required in each group. For possible dropouts, 50 participants in each group were enrolled in the study.

To determine whether the data were normally distributed, a Shapiro–Wilk normality test was applied. As most of the data were not normally distributed, data were presented in medians (interquartile ranges) and rates. Also, a non-parametric independent-samples t-test for was used. The χ^2^ or Fisher’s exact test was used when comparing the proportions between the groups. *P* values < 0.05 were considered significant. SPSS software (SPSS, Chicago, Ill) was used for the calculations.

## Results

We included 77 participants in the final analysis: 34 in the conventional group (classical simulation-based training) and 43 in the hybrid group. Both conventional and hybrid groups did not differ significantly in respect to gender, age, the ratio of students or residents (Table 1).

**Table 1:** Comparison of participants’ data between the conventional and hybrid groups

Gender n (%)	Conventional group (*n* = 34)	HybridLab group (*n* = 43)	p
Gender (female/male) (percentages)	23/11 (67/33%)	31/12 (72/28%)	0.803
Age (years)	22 (3)	22 (3)	0.526
Students/residents (percentages)	25/9 (74/26%)	30/13 (70/30%)	0.802

The results of the individual endotracheal intubation steps (16 actions) according to the checklist completed by the anesthesiologist in the OR with the subjects working with real patients are demonstrated in Table 2. Completion of all the actions of endotracheal intubation were significantly better performed by the subjects in experimental group when compared to those from the control group.

**Table 2:** Completion of 16 steps of endotracheal intubation comparison between conventional and hybrid groups. (ETT-endotracheal tube)

Action	Conventional group (n-34)	Hybrid group (n-43)	*P*
1. Checks equipment	56%	86%	0.003
2. Connects monitor	47%	91%	< 0.001
3. Preoxygenates correctly	27%	70%	< 0.001
4. Ensure correct patient position	77%	95%	0.019
5. Correct laryngoscopy	68%	95%	0.001
6. Describes laryngoscopy findings	71%	95%	0.003
7. Performs the right actions according to visual anatomical findings	62%	91%	0.002
8. Performs the right actions according to findings and situation	68%	95%	0.001
9. Correct insertion of the ETT (endotracheal tube)	47%	81%	0.002
10. Checks ETT depth	44%	93%	< 0.001
11. Inflates ETT cuff	65%	95%	0.001
12. Removes laryngoscope blade	56%	81%	0.015
13. Asks for CO_2_ detector and checks it	47%	95%	< 0.001
14. Auscultates chest to assess position of ETT	50%	93%	< 0.001
15. Secures ETT	32%	86%	< 0.001
16. Assess ETT cuff pressure	9%	58%	< 0.001
Overall			
Median (interquartile range)	53% (23%)	92% (13%)	< 0.001
Mean (standard deviation)	52% (18%)	88 (11%)	

The correct sequence of actions in the OR was compared between the groups. The hybrid group was 100% accurate with all 43 subjects, while the conventional group had only 11 of 34 subjects follow the correct actions sequence (32%) (*p* < 0.001).

The distribution of correctly performed endotracheal intubation actions in both groups is presented in Table 3. In the hybrid group the number of correct actions performed between 76 and 100% was 91%, while in the conventional group it was 32%.

**Table 3:** Distribution according to correctly performed endotracheal intubation skill actions between the conventional and hybrid groups

Actions performed correctly (approximate percentage)	Conventional group (*n* = 34)	Hybrid group (*n* = 43)	*P*
1 ⩾12 (~ > 76–100%)	11 (32%)	39 (91%)	< 0.001
2 11–8 (~ 51–71%)	9 (27%)	3 (7%)	
3 7–4 (~ 26–50%)	10 (29%)	1 (2%)	
4 ⩽ 3 (~ < 25%)	4 (12%)	0	

A survey of student confidence and satisfaction showed that students’ confidence to pass the final test did not differ significantly between the groups. However, the satisfaction was significantly higher in the hybrid group as compared to conventional one (Table 4).

**Table 4:** Comparison of students’ confidence and satisfaction survey results between the conventional and hybrid groups

Students’ survey		Conventional group (*n* = 22)	Hybrid group (*n* = 36)	*P*
Confidence question: 1. After learning, I think I will pass the test successfully.	5 (strongly agree)	4 (18%)	10 (28%)	0.259
	4 (agree)	6 (27%)	13 (36%)	
	3 (undecided)	8 (36%)	7 (19%)	
	2 (disagree)	4 (18%)	3 (8%)	
	1 (strongly disagree)	0 (0%)	3 (5%)	
Satisfaction question: 2. I enjoyed studying in this module.	5 (strongly agree)	3 (14%)	14 (39%)	0.02
	4 (agree)	5 (23%)	9 (25%)	
	3 (undecided)	8 (36%)	6 (19%)	
	2 (disagree)	2 (9%)	6 (17%)	
	1 (strongly disagree)	4 (18%)	1 (0%)	

## Discussion

The study demonstrated that a hybrid training method significantly improves the practical skill performance of endotracheal intubation as compared to the conventional simulation-based learning. We found that the overall average score of correctly performed endotracheal intubation procedure steps was on average 36% higher in the hybrid group than the conventional learner group. Similarly, other studies showed that simulation methods may improve training for acquiring practical skills [11–17]. This is in accordance with another study in which the hybrid training method was used. An overall average score was 96% of neonatal resuscitation skills among medical students [4] and in this study it was 88% of the endotracheal intubation skill. In the former study, the participants were evaluated by performing the skill on a manikin, while the current study allowed the subjects to apply the new skill on real patients. This suggests that hybrid training may allow achievement of similar learning results in both simulated and real clinical conditions.

When performing clinical skills such as endotracheal intubation, it can be said that there is little room for error; thus, perfect training requires an evaluation grade of 100%. Such high results were not achieved by either of our studied groups. One reason is that this was the first time that the participants had a chance to perform the intubation on real patients. However, the hybrid group performed perfectly on average 91% (varying between 76 and 100%) of the time, while in the conventional group it was only 32%. We believe that a better performance of the hybrid group could be attributed to the training in small groups of three (in comparison to 10–12 trainees per group in the classical training sessions) and the opportunity for each student to perform the skill many times at their preferred pace. The HybridLab setting allowed for repeated training sessions based on student preferences because they had 24/7 access to the skill lab and did not need to coordinate the time with an instructor or technician. In the classical simulation training model students and teachers alike are frequently restricted by time limits in face-to-face contact and the need to create equivalent learning opportunities for each student in the larger group due to differences in advance preparation and/or individuals’ capacity to acquire technical skills.

Peer-to-peer simulation sessions can be carried out at the pace needed by the learners and repeated until students develop automaticity and confidence; in the conventional class the instructor works with a larger group and clear time parameters, students are likely to have less hands-on time. Additionally, the better results might be due to the algorithm-driven and stepwise approach in HybdridLab training.

All participants in the HybridLab group followed the correct sequence of actions, while only 11 participants (32%) in the conventional group followed the correct sequence. The HybdridLab training being algorithm-driven and using a stepwise approach does not allow one to miss a single step during training. In this way, learning is based on 100% success, rather than making mistakes and reflecting on them after the procedure is completed. These findings are important, as patient safety necessitates a strict sequence of actions be adhered to in medical procedures [18, 19].

We found that subjects’ confidence about passing the final evaluation was not statistically different between the groups. However, satisfaction was significantly higher in the hybrid group. The hybrid learning has a well-prepared structure, good academic content and environment, and interactive practical training classes [4–6]. Overall, hybrid learning may increase the students’ satisfaction and motivation for learning and in this way improve the learning outcomes.

### Limitations

There are several limitations in our study. Firstly, we did not collect and are unable to present the patients’ data regarding airways difficulty and distribution between the groups. However, only ASA grade I and II patients with not difficult airways were selected prior intubation by one consultant who was blinded to student/resident randomization. Second, a larger dropout of participants was observed in a conventional group as compared to the hybrid group. The reason for this could be that the participants in the hybrid group were more motivated to complete the course as has been suggested previously [4–6].

## Conclusions

The findings support the theoretical model of mastery learning integrated with deliberate practice. The hybrid model broke the highly complex task of endotracheal intubation into smaller steps that the hybrid learners progressed through, adding new steps one at a time. This was supported by peer-to-peer feedback that improved performance. With adequate time provided to achieve mastery at each step before moving on to the next step, the hybrid group was able to build a level of performance that exceeded the conventional group, who are generally time- limited and lack ongoing individualized feedback needed for continuous improvement.

We conclude that novel algorithm-driven hybrid learning method improves the acquisition of the endotracheal intubation skills as compared to conventional simulation-based training with a high degree of acceptability and satisfaction of the learners.

## Data Availability

All data generated and analysed during the current study are available from the corresponding author on reasonable request.

## Abbreviations

ETI: Endotracheal intubation
LUHS: Lithuanian University of Health Science
OR: Operation room
ASA: American Society of Anesthesiologists
